# Structure-dependent interfacial behavior of bioinspired glycolipid surfactants

**DOI:** 10.1039/d6ra02903e

**Published:** 2026-07-21

**Authors:** Tyler J. Durkin, Kelsey R. Graves, Suchol Savagatrup, Raina M. Maier, David E. Hogan

**Affiliations:** a Department of Environmental Science, University of Arizona Tucson AZ 85719 USA davidehogan@arizona.edu; b Department of Chemical and Environmental Engineering, University of Arizona Tucson AZ 85719 USA; c School of Mining Engineering & Mineral Resources, University of Arizona Tucson AZ 85719 USA

## Abstract

Surfactants continue to find expanding applications in areas such as environmental remediation, consumer product formulation, and textile processing; however, their petrochemical origin and associated environmental toxicity remain significant concerns. As a result, considerable effort has been directed toward the development of “green” surfactants, which are naturally derived and exhibit reduced toxicity while maintaining the functionality of conventional surfactants. Glycolipids, a class of bacterially derived surfactants composed of a sugar headgroup and alkyl tail, represent a promising subset of these materials with potential in applications such as environmental remediation and wastewater treatment, agriculture, aqueous mining, cosmetics, and detergents. Traditionally, glycolipids are produced by *Pseudomonas aeruginosa* as mixtures of mono- and dirhamnolipids with alkyl chains containing 10 carbons. However, recent advances in synthetic chemistry have enabled the production of single congeners and systematic variation of both headgroup composition (*e.g.*, rhamno-, xylo-, and galactolipids) and alkyl chain length. The interfacial properties of these materials have not been comprehensively characterized. Here, we report a systematic evaluation of the surface and interfacial properties of a series of newly synthesized glycolipids. These compounds exhibit significantly lower critical micelle concentrations than sulfate surfactants of comparable alkyl chain length, while maintaining similar minimum surface tension values. This indicates that glycolipids retain comparable effectiveness while offering substantially greater efficiency. Variations in headgroup identity produce minimal changes in interfacial behavior, whereas alkyl chain length and tail architecture (single *vs.* double) have a pronounced impact. Together, these results elucidate structure–property relationships governing interfacial behavior and provide a foundation for the development of applications leveraging these green surfactants.

## Abstract 2

Synthetic glycolipid surfactants were systematically characterized. They exhibited lower critical micelle concentrations than sulfate surfactants with comparable surface tension, while alkyl chain structure strongly governed interfacial behavior.

## Graphical abstract

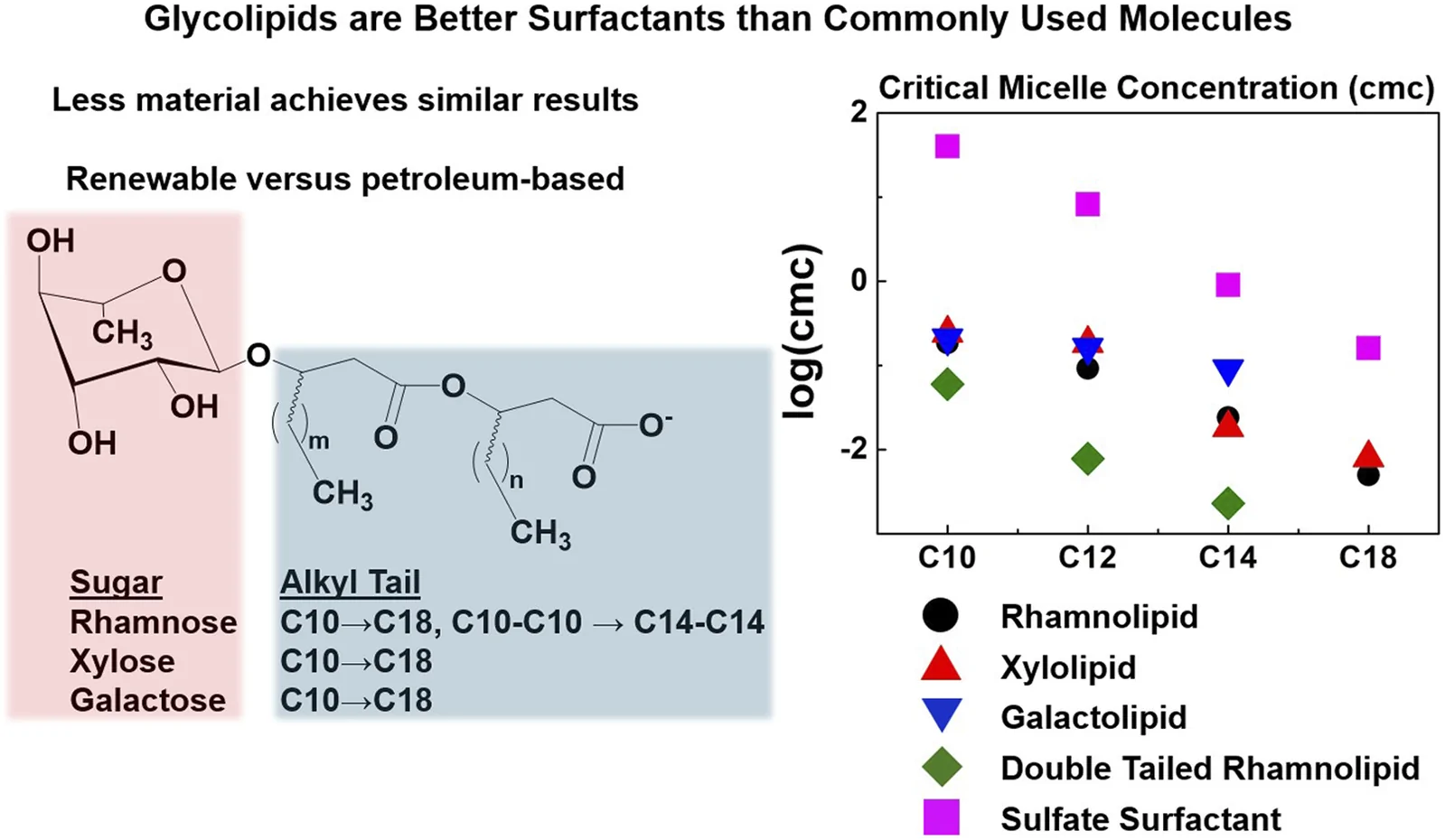

## Introduction

Surfactant molecules are increasingly utilized in a broad range of fields, including the fabrication of sensing devices, commercial product development (such as paints, adhesives, and detergents), environmental remediation of contaminated areas, agricultural sprays, textile processing, and enhanced oil recovery.^1–7^ The utility of these materials for such applications is due to their activity at interfaces that results from their amphiphilic structure. Surfactant structures are characterized by a hydrophilic ‘head group’ moiety (*e.g.*, sulfate, carboxylic acid, ammonium, *etc.*) paired with a hydrophobic ‘tail group’ moiety (*e.g.*, a lipid or similar alkyl chain).^6–8^ Surfactant structures vary greatly in complexity and composition, but common household materials include surfactants such as sodium dodecyl (lauryl) sulfate (SDS), sodium stearate, and alkylbenzene sulfonates.^9,10^ Such materials are typically petroleum-derived, and many of these surfactants are poorly biodegradable.^11,12^ Furthermore, some are classified as toxic and may pose risks to human (respiratory, kidney, or gastrointestinal concerns) and environmental health.^13–16^ As a result, the use of these surfactants in both existing and new applications is under increased scrutiny.^17–19^

It is estimated over 15 million tons of various surfactants are used worldwide each year, with upwards of 60% ending their life dispersed in the aquatic environment.^20,21^ Here, they can interact with organic and metal contaminants, contributing to system-wide toxicity and eutrophication, and compromise the integrity of ecosystems.^20^ To mitigate the concerns associated with petrochemically-derived surfactants, so-called “green” surfactants, which are comprised of non-toxic components such as sugars, lipids, and other similar sustainable compounds, have emerged as alternative materials.^20,22–26^ With a broad spectrum of green surfactant molecules in existence, they can be broadly categorized into groups such as sophorolipids, surfactins, mannosylerythritol, and glycolipids.^27^ Importantly, green surfactants exhibit the same functional properties that render standard surfactants desirable for the aforementioned applications (*e.g.*, capacity for interfacial absorption, emulsification, foaming, and anti-corrosive properties) and have been observed to reduce surface and interfacial tension to comparable or greater levels than those standard surfactants.^26,28^ Furthermore, green surfactants have already been successfully incorporated into applications like pharmaceuticals (drug delivery), agriculture, and cosmetics. Knowing this and given their favorable characteristics and minimal impact on the environment and human health, these green surfactants are positioned as leading candidates for next-generation surfactant design.

Glycolipids, briefly mentioned above, are a recognized subcategory of green surfactants that have unique surfactant properties and are of interest as potential replacements for traditional petrochemically derived surfactants.^29–31^ The structure of a glycolipid mimics a standard surfactant with a sugar molecule as the hydrophilic head group and a lipid between 8 and 18 carbon atoms as the hydrophobic tail, typically a hydroxy alkanoic acid, such as 3-hydroxydecanoic acid. Among glycolipids, the rhamnolipids have historically been the most well-researched. Their head group contains one or two rhamnose sugars and the tail-group contains either one or two (3-(3-hydroxyalkanoyloxy) alkanoic acid) molecules. Rhamnolipids were first described as secondary metabolites from *Pseudomonas aeruginosa* and now more than 110 rhamnolipid congeners from a variety of microorganisms have been described.^32–35^ Rhamnolipid is produced as complex mixtures consisting of single and double rhamnose head groups and tail groups varying in number, length, and saturation.^35^ Though the overall mixture of rhamnolipids produced is fully strain specific, in practice, it has been observed that most *P. aeruginosa* strains predominantly produce (between 70-80%) l-rhamnosyl-β-hydroxydecanoyl-β-hydroxydecanoate and l-rhamnosyl-l-rhamnosyl-β-hydroxydecanoyl-β-hydroxydecanoate rhamnolipids, henceforth referred to as Rha-C10-C10 and Rha-Rha-C10-C10, respectively.^36,37^

Previous research has characterized the properties and potential applications of these bacterially produced rhamnolipids. They can reduce the surface tension of the air–water interface to approximately 30 mN m^−1^ from the standard 72 mN m^−1^ even at comparatively low concentrations, a hallmark of an effective surfactant.^38–40^ Additional research has worked to understand the process of absorption of rhamnolipids to fluid interfaces (*e.g.*, diffusion, formation of micelles in the bulk, *etc.*).^41,42^ Based on these characteristics, rhamnolipids have been widely investigated for applications in environmental remediation of hydrocarbons and metals, cosmetics, personal care and household products, food and agriculture, antimicrobial applications, and biotechnology.^43–46^

The large number of congeners made biologically and the difficulty in producing consistent congener mixtures from batch to batch led to the need to produce high purity single congener rhamnolipids to advance their broad commercial use. Using green chemistry approaches, it is now possible to produce single rhamnolipid congeners.^40,47–50^ Further, these synthetic methods allow for the production of glycolipids not known in nature. Indeed, it is possible to synthesize a glycolipid with a xylose (Xyl) or galactose (Gal) headgroup in place of rhamnose (Rha) and to control both the length and number of alkyl tails in the molecule.^47^ In contrast to biosynthesized rhamnolipids which produce a single diastereomer, synthetic production results in four diastereomers for double-tailed rhamnolipids and two diastereomers for single-tailed rhamnolipids. These seemingly simple changes can result in large differences in the interfacial properties of glycolipids due to their effects on the amphiphilicity and hydrophilic/hydrophobic balance of the molecules. To this point, the interfacial characteristics of these newly available glycolipids have not been analyzed in detail. We contend that measuring these values is imperative in the determination of relevant applications (environmental, commercial, biological, *etc.*).

This work presents a comprehensive examination of the structure-dependent interfacial properties of newly synthesized glycolipids (rhamno-, xylo-, and galactolipids) of alkyl tail lengths between 8 and 18 carbon atoms (both single and double-tailed). We provide an analysis of critical micelle concentration and minimum surface tension to compare the overall surfactant effectiveness of each individual glycolipid. We then use this experimental data as a basis to compare the surfactant performance of the glycolipids. As assembly at the air/water interface can be considerably different than assembly at hydrocarbon/water interfaces, we next worked to define the interfacial tension of a representative hydrocarbon (toluene)/water interface in the presence of the various glycolipids. Thus, the overall aim of this study is to elucidate these fundamental properties to support the use of these glycolipids as “green” surfactants *in lieu* of conventional surfactants in applications such as environmental remediation and wastewater treatment, agriculture, aqueous mining, cosmetics, and detergents.

## Experimental

### Choice of surfactants and materials

**Fig. 1:**
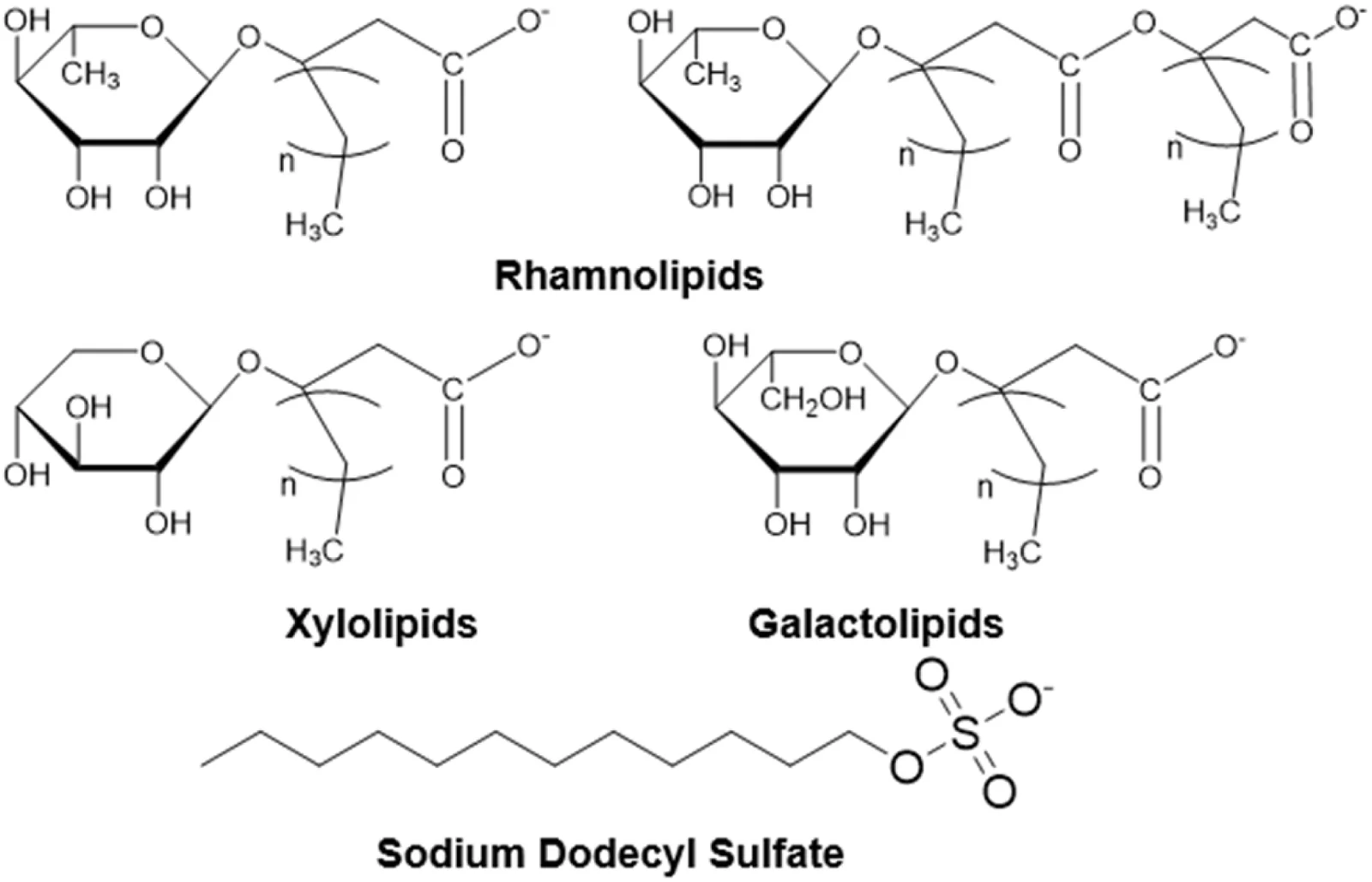
General chemical structure of rhamno-, xylo-, and galactolipids used in this study (*n* = 6, 8, 10, or 14, depending on the specific molecule in question) compared to the structure of the common synthetic surfactant, sodium dodecyl sulfate.

We selected a variety of synthetic, bio-inspired glycolipids to examine the potential effects of structure on interfacial activity (Fig. 1). Glycolipids with rhamnose (Rha), xylose (Xyl), and galactose (Gal) sugar head groups were selected with fully saturated alkyl tails. We selected an array of different single tail lengths in conjunction with each of the sugar heads to provide both short chain glycolipids (tails of 10 and 12 carbon in length) and long chain glycolipids (tails of 14 and 18 carbon in length). In conjunction with single-tailed glycolipids, we were also interested in the variance in interfacial behavior between the single- and double-tailed molecules. Thus, we examined double-tailed rhamnolipids with symmetrical tails of 10, 12, and 14 carbons in length. Surfactants containing C18–C18 tails were not evaluated due to their limited solubility in aqueous solutions. We also chose sodium dodecyl sulfate (SDS) as an example of a traditional, petrochemically derived surfactant for comparison purposes; the interfacial behavior and properties of SDS are well-understood, and as such, it presents an attractive model for comparison with new molecules whose behavior is more uncertain.^51^ Furthermore, SDS, like the studied glycolipids, is an anionic surfactant, which allows for charge on the surfactant molecules to remain a constant throughout the study. All glycolipid surfactants were purchased from GlycoSurf, Inc. (Salt Lake City, UT), while SDS and toluene were purchased from Sigma-Aldrich. All chemicals were used as received without further purification. Surfactant solutions were prepared by dissolving the desired concentration of glycolipid in ultrapure water (resistivity of ≥18.2 MΩ cm).

### Measurement of interfacial properties (surface and interfacial tension)

To characterize the glycolipids' interfacial properties, *i.e.*, surface and interfacial tension, we used two tensiometric methods. First, a force tensiometer equipped with a platinum–iridium (Pt–Ir) Du Noüy ring as the probe was used to determine critical micelle concentration and minimum surface tension.^52^ In force tensiometry, surface tension is directly related to the tensile force measured at the interface the ring traverses. Platinum (or gold) is chosen for construction of the ring to eliminate any potential effects of imperfect contact between the probe and the aqueous phase (*i.e.*, contact angle > 0°), and the tensile force of a lamella beneath the ring is measured and corrected through the Harkins–Jordan method. To ensure precision, all measurements were triplicated in separate sample vessels. Each vessel was thoroughly cleaned with deionized water, isopropyl alcohol, and flame after each trial to ensure no residual surfactant was present. All surface tension values were recorded at room temperature (∼20 °C).

Second, we used pendant drop tensiometry with the Young–Laplace equation, a subclass of droplet-based tensiometry in which a droplet of one fluid (the droplet phase) is completed surrounded by the other (the continuous phase). In this approach, the fluid itself serves as the probe instead of a separate ring or plate as is done in force tensiometry. This measurement technique allows for both surface tension measurements (a liquid suspended in air) and interfacial tension (liquid in liquid). For these surface tension measurements, we maintained the droplet volume at 10 µL as larger droplets are inherently unstable in air and lead to large variations in measured surface tension. As the pendant drop method is dynamic and measures surface tension in real-time, we collected data over a 5-minute period, which allows for the system to completely equilibrate. To allow a comparison of surface tension collected by this method to force tensiometry, we record this equilibrium surface tension (*i.e.*, the average of the final 20 seconds of data).

Two tensiometric approaches were used as we speculated that the critical micelle concentration of the short-chain glycolipids would be larger than their long-chain equivalents, as has previously been reported for synthetic surfactants.^53,54^ Force tensiometry requires a large amount of solution (potentially on the order of hundreds of milliliters), and consequently, surfactant, to produce an accurate reading of minimum surface tension. Alternatively, the pendant drop method allows for the use of smaller quantities of material to obtain a reading of minimum surface tension which is important when overall quantities of surfactant are limited or cost is prohibitively high.^55^ These two approaches were used to conserve material, minimize solution volumes, and facilitate measurements within surfactant concentration regimes best suited to each method. The approach used for each glycolipid is reported in Table 1 below.

**Table 1:** Interfacial properties of the studied glycolipids: critical micelle concentration, minimum surface tension, and interfacial tension. Measurements were obtained using pendant drop tensiometry unless otherwise noted

Head group	Alkyl tail length	Critical micelle concentration (mM)	Minimum surface tension (mN m^−1^)	Interfacial tension (mN m^−1^)
Rhamnose	C10	0.185	30.21 ± 0.78	23.23 ± 0.57
	C12	0.092	32.35 ± 0.84	26.89 ± 0.80
	C14	0.024	33.16 ± 0.42	26.21 ± 1.16
	C18	0.005^a^	33.90 ± 0.55^a^	18.86 ± 3.25
	C8–C8	0.098	25.95 ± 0.22	9.82 ± 0.69
	C10–C10	0.006	25.73 ± 0.30	17.45 ± 0.98
	C12–C12	0.008	25.63 ± 0.04	17.95 ± 0.87
	C14–C14	0.002	26.19 ± 0.08	20.47 ± 0.48
Xylose	C10	0.242	33.20 ± 0.85	21.86 ± 0.36
	C12	0.182	33.23 ± 0.35	26.08 ± 1.35
	C14	0.018	32.34 ± 0.63	21.63 ± 0.15
	C18	0.008^a^	33.99 ± 0.66^a^	16.01 ± 2.76
	C10–C10	0.087	25.22 ± 0.09	—
Galactose	C10	0.211	33.25 ± 0.91	27.12 ± 0.56
	C12	0.163	31.46 ± 0.70	24.97 ± 0.24
	C14	0.091	31.32 ± 1.04	22.98 ± 0.93
	C18	—	—	22.22 ± 0.66
	C10–C10	0.110	24.98 ± 0.05	14.02 ± 0.32
Sulfate	C10	10.62	40.89 ± 0.85	—
	C12	8.201	31.60 ± 0.62	21.44 ± 1.01
	C14	1.712	39.73 ± 1.42	—
	C18	0.174	36.35 ± 0.33	—

a Data were collected by force tensiometry.

In all experiments for the measurement of interfacial tension, we selected a toluene and ultrapure water interface as our standard and used pendant drop tensiometry in conjunction with the Young–Laplace equation. The toluene/water interface is well-characterized without the presence of surfactant molecules and has a known interfacial tension (∼32–37 mN m^−1^).^56^ We used an inverted “J” hook to form the droplets in an upside-down configuration because toluene is less dense than water. Furthermore, the glycolipids in question are not observed to be soluble in toluene, ensuring that the surfactant remains at the interface or in the bulk aqueous solution and does not partition to the hydrocarbon oil phase. To ensure consistency between individual trials, we maintained a constant droplet volume of 25 µL as well as a constant volume of 5 mL of the aqueous surrounding phase. Experiments were performed in triplicate and the results averaged to produce a singular reported data point.

### Estimation of relevant parameters

To better understand the behavior of the glycolipid molecules in solution and how they would orient on a molecular level at air/water or water/oil interfaces we estimated both the hydrophilic–lipophilic balance (HLB) and the critical packing parameter (CPP) of each studied glycolipid. HLB according to the Griffin Method is a value between 1 and 20 that indicates whether a surfactant is more “water-loving” or “water-hating” where larger numbers correspond to more hydrophilic molecules. This numeric quantity is calculated by simply dividing the hydrophilic weight percent of the overall molecule by 5. The critical packing parameter is a dimensionless geometric ratio that helps to predict the preferential micellar structure (spherical, cylindrical, *etc.*) of surfactants in solution. This estimation is achieved using Tanford's equations to define tail length and volume and then relating these calculated parameters to the head group area.^57^

## Results and discussion

Table 1 contains a full spectrum of the interfacial data collected in this study and provides insight into the role of glycolipid structure in surfactant activity. To elaborate, we present critical micelle concentration (CMC), minimum surface tension (MST), and interfacial tension of a toluene/water interface at a set concentration of glycolipid for a series of rhamnolipids, xylolipids, and galactolipids. Specific trends will be discussed in detail below, but briefly, varying the head group of the glycolipids does not significantly alter surfactant properties apart from interfacial tension. Clear differences arise from variation in the alkyl tail length with increased tail length resulting in decreased CMC. This effect is further pronounced in glycolipids with two tails which exhibited lower CMCs as well as surface and interfacial tensions. Within these results, glycolipids are compared using the terms effectiveness and efficiency, which refer to different surfactant properties. Surfactant effectiveness is defined as the MST achieved at the CMC. In contrast, efficiency refers to the amount of surfactant required to reach a defined point, such as the CMC. Therefore, when comparing two surfactants, a more efficient surfactant reaches its CMC at a lower concentration, but it may be more/less effective if it achieves a lower/higher MST, respectively, than the other.

Building upon this, Table 2 details the estimated HLB and CPP of each studied glycolipid. These results aid in the understanding of how differences in glycolipid chemistry and structure impact the physical behavior of the molecules in solution. Specifically, increasing the chain length of the alkyl tail increases the hydrophobicity of the glycolipids, which is expected. Importantly, we note that typical water-soluble surfactants fall within an HLB range of 8–18, which encapsulates the vast majority of the studied glycolipids, including all of the single-tailed glycolipids and the shorter-chain double-tailed surfactants. Indeed, these glycolipids do maintain water solubility. The longer double-tailed rhamnolipids fall into the moderate HLB category, indicating that these materials are more hydrophobic in nature (as expected). These results when taken together indicate that HLB index is far more influenced by the tail length of the surfactant than varying the headgroup.

**Table 2:** Calculated values of the hydrophilic–lipophilic balance (HLB) and critical packing parameter (CPP) of each studied glycolipid

Head group	Alkyl tail length	Hydrophilic–lipophilic balance (HLB)	Critical packing parameter (CPP)
Rhamnose	C10	11.48	0.40
	C12	10.59	0.40
	C14	9.83	0.40
	C18	8.60	0.41
	C8–C8	9.81	0.40
	C10–C10	8.72	0.41
	C12–C12	7.85	0.41
	C14–C14	7.14	0.41
Xylose	C10	11.12	0.48
	C12	10.22	0.48
	C14	9.46	0.48
	C18	8.23	0.48
	C10–C10	8.40	0.49
Galactose	C10	11.88	0.50
	C12	11.01	0.50
	C14	10.24	0.50
	C18	9.00	0.50
	C10–C10	9.07	0.50

In contrast, the packing parameter of studied glycolipids has been found to be far more affected by the head group identity than the tail length. Indeed, the individual categories of glycolipids are largely identical in packing parameter amongst each type (rhamno-, xylo-, and galactolipids). Rhamnolipids as a subcategory display the smallest packing parameter of the trio, which stems from the fact that it is the largest headgroup, a metric which is inversely proportion to packing parameter. Indeed, rhamnolipids likely form cylindrical micelles in solution due to this smaller CPP. Both xylolipids and galactolipids, with larger packing parameters can potentially assemble into vesicles or bilayers. These differences in CPPs help to provide insight into interfacial behavior and consequently, can be used to understand trends in surface and interfacial tension.

### Characterization of single-tailed glycolipids

We began by examining the effect of glycolipid concentration on surface tension to identify CMC and MST. Within each trial, the concentration of the surfactant was varied and only one glycolipid type was present in each tested solution. We note that while we were able to work with Rha-C18 and Xyl-C18, we were unable to solubilize Gal-C18 at any relevant concentration for surface tension analysis and thus were unable to determine its CMC and MST.

**Fig. 2:**
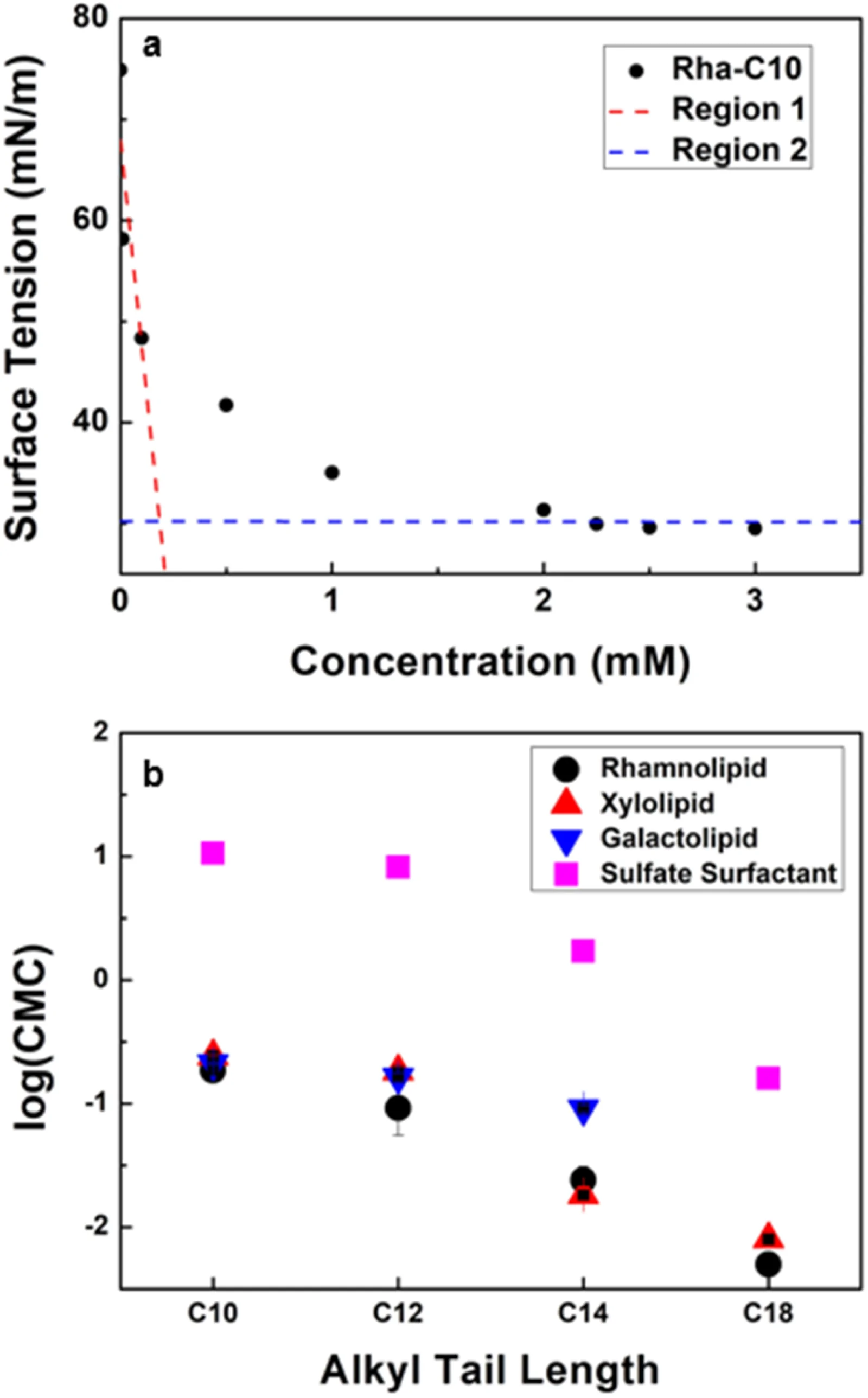
(a) Representative surface tension isotherm used to calculate CMC and MST of glycolipids, in this case Rha-C10. The CMC of the tested surfactant occurs at the intersection between the lines indicating “Region 1” and “Region 2”, while the MST is the *y*-intercept of the Region 2 line. (b) Stauff–Klevens plot of rhamno-, xylo-, and galactolipids compared to sodium alkyl sulfate surfactants.

Fig. 2a demonstrates an example of the surface tension/concentration plots (in this case, for Rha-C10) used to determine CMC and MST. Briefly, to calculate CMC graphically, surface tension is measured at a wide variety of surfactant concentrations, and the CMC is the extrapolated intersection of the final plateaued baseline and the sharply decreasing start-up of the concentration gradient plot, as shown in “Region 1” and “Region 2” above. Fig. 2b demonstrates an example of a Stauff–Klevens plot for the full array of analyzed glycolipids as well as standard sodium *n*-decyl sulfate surfactants with identical alkyl tail lengths to the glycolipids, for comparison.^58^ As observed in Fig. 2, each of the surfactants studied follows the trend expected as per standard Stauff–Klevens analysis: CMC linearly decreases with increasing alkyl tail length on a logarithmic scale. Indeed, we found that shorter chain surfactants have higher CMCs than their longer chain counterparts, though all CMC values are on the order of micromoles. As a whole, the glycolipids are more efficient than sulfate surfactants with equivalent alkyl tail lengths (*e.g.*, sodium decyl, dodecyl, tetradecyl, and octadecyl sulfate). This is particularly stark for the shorter-chain glycolipids as they are orders of magnitude more efficient than sulfate surfactants, which is particularly relevant in applications in which minimal total concentration of surfactant is desired. For example, the CMC of C10 sulfate is 10.62 mM while values range from 0.185 to 0.242 mM for the C10 glycolipids.

These findings align with previous studies of chain variation in standard molecular surfactants. In general, for surfactants, increasing the chain length of surfactants has been observed to decrease the CMC and MST. As a specific example, Evans noted that the CMC of sulfate surfactants with alkyl tails between 8 and 18 carbon atoms in length decreases exponentially with increasing tail length.^59^ With similar sugar-based surfactants as the glycolipids used herein, Marchant and colleagues similarly found that increasing the alkyl tail length of a variety of saccharide surfactants results in a decrease in the CMC of the surfactants by a factor of 10.^60^

**Fig. 3:**
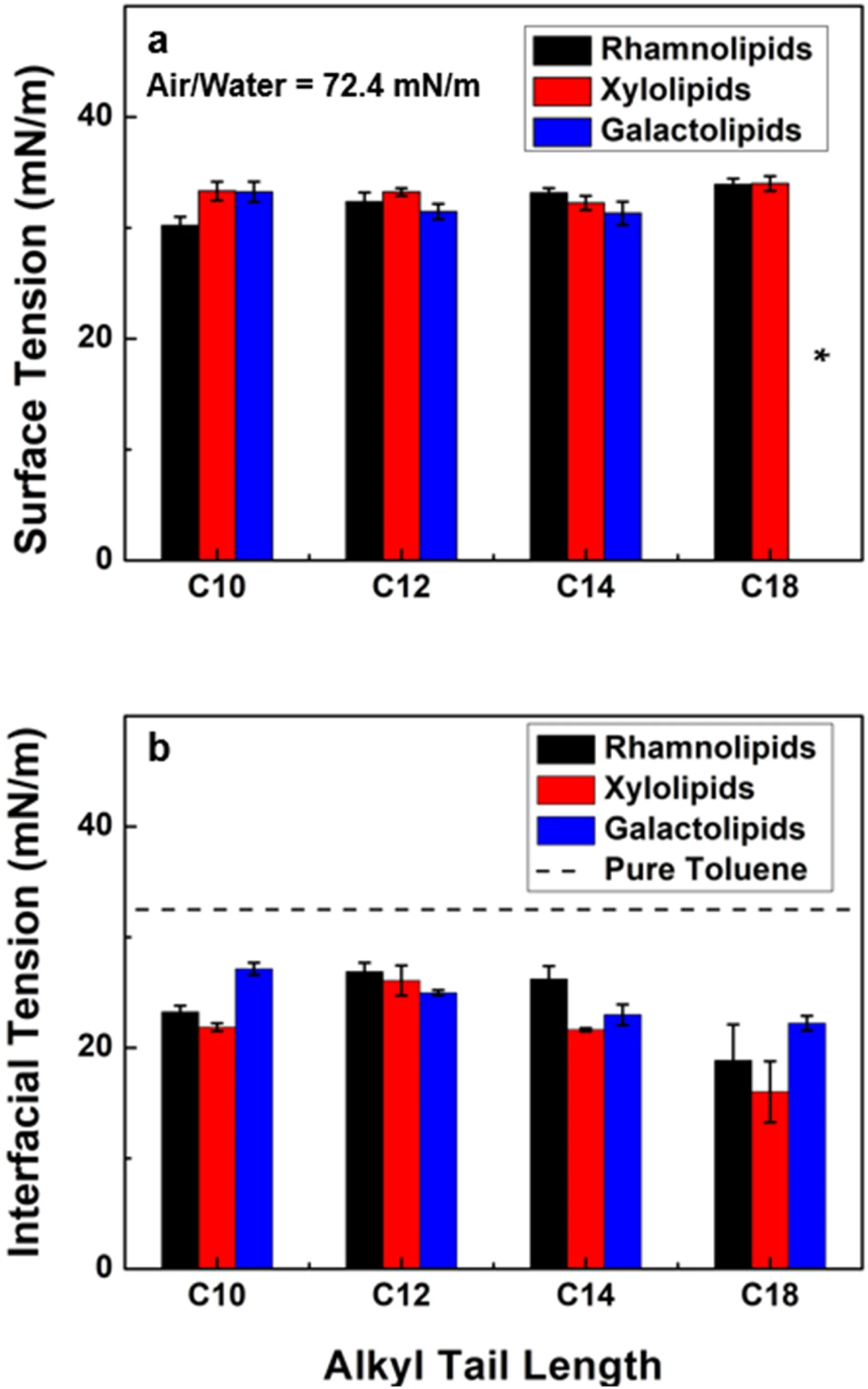
(a) MST of studied glycolipids organized by alkyl tail length. (b) Interfacial tension of a toluene/water interface in the presence of 0.01 mM of glycolipid surfactant.

Contrasting to CMC data, examination of the single-tail glycolipids showed MST was consistent across the series (Fig. 3a). This indicates these glycolipids are similarly effective regardless of tail length. MST for each single-tailed glycolipid was recorded between approximately 30–33 mN m^−1^; this is comparable to the MST of SDS and other, comparable molecules which have been found to be in similar regimes.^61^ Taking a closer look in conjunction with knowledge of CMC, the short-chain rhamnolipids are the best single-chain glycolipids, as they possess the lowest CMC and a similar MST when compared to the xylo- and galactolipids. With longer-chain glycolipids, Xyl-C14 and Rha-C14 have lower CMCs (0.018 and 0.024 mM, respectively) than that of Gal-C14 (0.091 mM) while all three glycolipids have similar MSTs, leading to the selection of Rha-C14 and Xyl-C14 as the better choices. Valentini *et al.* also examined Rha-, Xyl-, and Gal-C14 and reported CMCs of 1.3 mM, 1.7 mM, and 3.3 mM, respectively, compared to our values of 0.024 mM, 0.018 mM, and 0.091 mM, respectively. While the overall values differ, our measurements were taken at room temperature while those of Valentini and coworkers were taken at 45 °C. As CMC is known to increase at an exponential rate at higher temperatures due to the inhibition of micelle formation, this variance is expected. Furthermore, the results are in agreement in that the CMCs of Rha-C14 and Xyl-C14 are both notably lower than Gal-C14. These results follow previously understood principles of glycolipids that dictate fewer hydroxyl groups in the sugar head lead to lower CMC values.^62,63^ Additionally, these observations align with the CPPs of each glycolipid, specifically, the more inefficient packing of galactolipids likely contributes to the higher observed CMCs.

To further characterize the structure-dependent behavior of these materials, we examined the glycolipids' activity at the interface of a toluene/water system using the pendant drop method. Interfacial behavior is particularly important for certain applications in which ultra-low interfacial tension values (*i.e.*, lower than 1 mN m^−1^) need to be achieved, such as for enhanced oil recovery, cleansing organic materials from both solid surfaces and aqueous environments, forming emulsions, or for various methods of liquid–liquid extractions. Interfacial tension of a hydrocarbon/water interface, in general, will be lower than that of surface tension of an air/water interface, but we can expect that trends in interfacial behavior will be similar in both cases. We measured the interfacial tension of a model toluene/water interface in the presence of the glycolipid surfactants at a constant glycolipid concentration of 0.01 mM. We note that as we used a considerably lower total concentration of glycolipids for these studies, we were able to explore glycolipids with limited solubility that were not amenable to surface tension analysis (*i.e.*, Gal-C18).

As shown in Fig. 3b, the glycolipids show greater variability in interfacial tension compared to surface tension (Fig. 3a). Though the majority of the glycolipids followed the same trends observed for MST, there are a few interesting trends of note that do not align with our expectations. Considering the single-tailed rhamno-, xylo-, and galactolipids, we hypothesized that increasing the chain length of the glycolipid would monotonically decrease the interfacial tension provided that total surfactant concentration remained unchanged, as was seen in surface tension. This trend has previously been observed through both molecular dynamic simulations and experimental observations of the behavior of common synthetic surfactants. One such observation was made by Smit and coworkers who demonstrated that with an increase in the length of the hydrophobic tail of a sodium *p*-(*x*-alkyl)benzenesulfonate surfactant from 9 to 12 carbons, the resulting interfacial tension of a decane/water interface decreases sequentially.^58^ A similar decrease in interfacial tension is observed within each sugar series of the single-tailed glycolipids as tail lengths increased from C12 to C18. However, for the Rha- and Xyl-C10 molecules, the interfacial tension was lower than that observed for the C12 molecules. To elaborate further, the addition of 0.01 mM Rha-C10 to the toluene/water interface reduced the interfacial tension by 4.74 mN m^−1^ while addition of Rha-C12 reduced by only 1.08 mN m^−1^. Xyl-C10 was even more effective, lowering interfacial tension by 6.11 mN m^−1^, while the reduction by Xyl-C12 was 1.88 mN m^−1^ (Fig. 3b).

These results contrast those observed at the same concentration of Gal-C10, which only reduced interfacial tension by 0.84 mN m^−1^. This discrepancy may be explained by galactolipids possessing the largest estimated packing parameter of any of the glycolipids, indicating that the surfactant may naturally orient in micelles instead of building up at the interface. Building upon this, we note that the Rha- and Xyl-C10 molecules should demonstrate superior interfacial packing and with their smaller size, can partition to the interface with greater speed, potentially accounting for this phenomenon. Due to this unique behavior in Gal-C10, the galactolipids follow the expected monotonic decrease in overall interfacial tension, while the rhamno- and xylolipids show minimal changes in interfacial tension across the C12 and C14 variants. We speculate that these unique trends may occur as a result of the formation of micelles in the bulk phase limiting interfacial interaction (and consequently, a smaller reduction in interfacial tension) in these “mid-length” surfactants.^64,65^ Furthermore, as noted from surface tension studies, at these concentrations of C18 surfactants, we have already reached CMC and thus, are observing the maximum reduction in interfacial tension possible using these materials.

### Characterization of double-tailed rhamnolipids

**Fig. 4:**
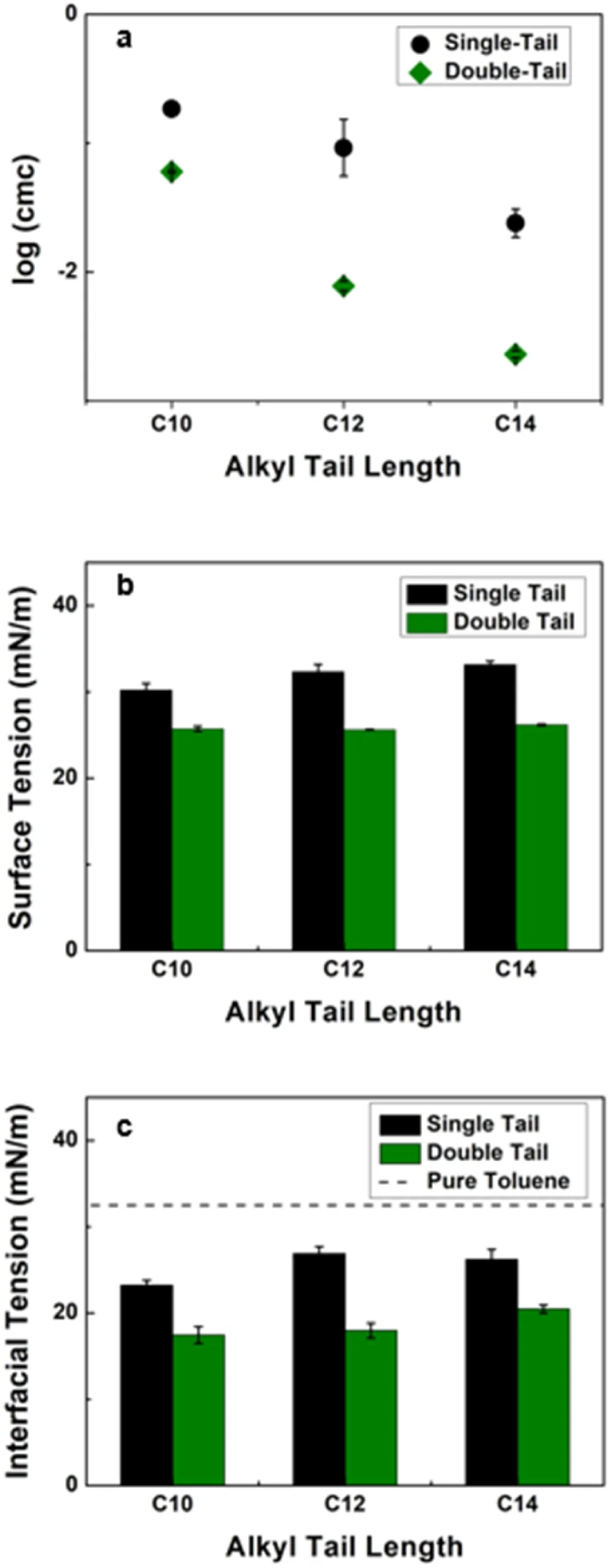
(a) Stauff–Klevens plot comparing the CMCs of single and double-tailed rhamnolipids at equivalent alkyl tail lengths. (b) MST of single and double-tailed rhamnolipids at equivalent alkyl tail lengths. (c) Interfacial tension of a toluene/water interface in the presence of 0.01 mM of single or double-tailed rhamnolipid.

In addition to single-tailed glycolipids, double-tailed rhamnolipids were evaluated to elucidate the effect of adding another hydrophobic tail to the molecule. It is shown that hydrophobic interactions drive surfactant self-assembly at the interface and attaching an additional alkyl tail leads to improved self-assembly.^66–68^ Therefore, adding an additional tail to the glycolipid structure should yield more effective and efficient surfactants than their single-tailed counterparts.^66–68^ As shown in Fig. 4a, the CMC of each the double-tailed rhamnolipids is considerably lower than each of their single-tailed analogs, which is expected due to their more hydrophobic nature. Importantly, with increasing alkyl tail length, the CMC of double-tailed rhamnolipids monotonically decreases, as expected with a standard Stauff–Klevens relationship. In conjunction with CMC data, we recorded MST, as shown in Fig. 4b. Indeed, double-tailed rhamnolipids have been found to be more effective surfactants than their single-tailed counterparts and were able to reduce surface tension to approximately 25 mN m^−1^, an increase of more than 5 mN m^−1^ from each of the single-tailed molecules studied. This trend holds with each of the chain lengths studied as well. Pitt and coworkers had similar findings that adding additional tails of equivalent length to surfactant molecules reduces both the MST and CMC of the system (to a point).^69^ Interestingly, the MST measured for Rha-C10-C10, C12–C12, and C14–C14 are relatively identical, suggesting that ∼25 mN m^−1^ may be the maximum potential surfactant effectiveness of molecules based on this glycolipid framework when using symmetrical tails. In a study using rhamnolipids, Palos Pacheco *et al.* demonstrated that varying the symmetry of the tail lengths in rhamnolipids with twin tails impacts the aggregation behavior of the molecules. Specifically, highly asymmetric rhamnolipids tend to preferentially form micelles due to the comparatively large size of the headgroup, while longer-tailed, more symmetric rhamnolipids tend to aggregate, leading to non-systemic changes in interfacial behavior.^70^ Both the CMC and MST of the asymmetric rhamnolipids were found to be generally lower (approximately an order of magnitude in CMC and 4 mN m^−1^ in MST) than their symmetric counterparts. Thus, it is further possible to tailor the interfacial behavior of these molecules by not only type and number of tail groups, but also the symmetry of the tails.

Study of interfacial tension of the double-tailed rhamnolipids revealed similar behavior to surface tension. Fig. 4c contains the recorded interfacial tension of a toluene/water interface with the addition of 0.01 mM of the specified glycolipid. As is shown, Rha-C10-C10 and Rha-C12-C12 reduce the interfacial tension to approximately 18 mN m^−1^, a considerable reduction from an identical concentration of Rha-C10 and Rha-C12, respectively. Rha-C10-C10 reduces the interfacial tension by 5.78 mN m^−1^ more than Rha-C10, and Rha-C12-C12 lowers the interfacial tension by an additional 8.94 mN m^−1^ compared to Rha-C12. We attribute these changes to the smaller CMC (0.006 mM, 0.008 mM, and 0.002 mM for Rha-C10-C10, C12–C12, and C14–C14, compared to 0.185 mM, 0.092 mM, and 0.024 mM for the single-tailed versions) and thus, greater surfactant efficiency of double-tailed surfactants. Though we observed a similar decrease in interfacial tension when using Rha-C14-C14, the overall interfacial tension of the system is larger than the other cases. The minimum interfacial tension reached is 20.47 mN m^−1^, which is approximately 2.5 mN m^−1^ higher than that observed for the other double-tailed rhamnolipids. This odd behavior may be connected to the greater hydrophobicity of the longer chain rhamnolipid resulting preferential formation of micelles. Overall, the double-tailed rhamnolipids, with both surface and interfacial tension, are superior options for surfactant efficiency and effectiveness compared to their single-tailed counterparts. This is especially enlightening as the two chain molecules with shorter chains are preferentially produced by microorganisms, suggesting an evolutionary optimization between interfacial activity and cellular resource investment.

Within the observed trends in CMC, it becomes relevant to consider the potential effects of stereochemistry within both the single and double-tailed glycolipids, as our studied glycolipids are not stereospecific. Specifically, it can be speculated that differences in molecular orientation between *S* and *R* configurations could lead to changes in the interfacial packing of the glycolipids. Palos Pacheco and coworkers investigated this potential concern by measuring the surface tension of stereospecific variations of Rha-C10-C10. The group found that while *R*,*R* and *S*,*R* diastereomers were packed far more loosely at the interface, there were no significant changes in the minimum surface tension of the molecules.^40^ Furthermore, CMCs of each of the tested rhamnolipids were consistent, with differences only observed under basic conditions. These results suggest that interfacial properties are not particularly affected by the stereochemistry of the glycolipids.

## Conclusions

In conclusion, we report a comprehensive interfacial characterization (critical micelle concentration, minimum surface tension, and interfacial tension at a set concentration) of a range of novel synthetic glycolipids. These include single-tailed rhamno-, xylo-, and galactolipids with alkyl chain lengths of C10–C18, as well as double-tailed rhamnolipids with symmetrical chain lengths of C10–C14. All analyzed glycolipids exhibit significantly lower CMC than sulfate surfactants with equivalent alkyl chain lengths, indicating superior efficiency relative to common petrochemical surfactants. Consistent with established trends in surfactant systems, increasing alkyl chain length leads to lower CMC values, while minimum surface tension and interfacial tension remain comparatively similar across chain lengths. In addition, double-tailed glycolipids are, overall, both more effective and efficient than their single-tailed counterparts. Together, these results elucidate structure–property relationships governing interfacial behavior and represent an important step toward the development of applications leveraging these green surfactants.

## Author contributions

Tyler J. Durkin: writing – review and editing, writing – original draft, investigation, conceptualization, formal analysis, conceptualization. Kelsey R. Graves: investigation, conceptualization Suchol Savagatrup: writing – review and editing Raina M. Maier: writing – review and editing, supervision, project administration, funding acquisition, conceptualization. David E. Hogan: writing – review and editing, supervision, project administration, funding acquisition, conceptualization.

## Conflicts of interest

The authors declare the following financial interests/personal relationships which may be considered as potential competing interests: David E. Hogan and Raina M. Maier report equipment, drugs, or supplies was provided by GlycoSurf, Inc. David E. Hogan reports a relationship with GlycoSurf, Inc. that includes: former employment. Raina M. Maier reports a relationship with GlycoSurf, Inc. that includes: board membership and equity or stocks. Raina M. Maier has patent #US9499575B2; WO2014077960A1; US20210403499A1 licensed to GlycoSurf, Inc. The terms of this arrangement have been reviewed and approved by the University of Arizona in accordance with its policy on objectivity in research.

## Supplementary Material

RA-016-D6RA02903E-s001

## Data Availability

Data supporting this manuscript is available in the supplementary information (SI). Additional data will be made available upon request. Supplementary information: additional plots detailing the calculation of critical micelle concentration for each glycolipid studied. See DOI: https://doi.org/10.1039/d6ra02903e.
