# Supplementary material for: Structure-dependent interfacial behavior of bioinspired glycolipid surfactants
Source: RSC Adv. 2026 Jul 21;16(38):42299–309. doi: 10.1039/d6ra02903e (PMC13387384; doi:10.1039/d6ra02903e)
Supplement: Supplementary file 1 [file RA-016-D6RA02903E-s001.pdf]

Supplementary Information (SI) for RSC Advances.
This journal is © The Royal Society of Chemistry 2026

Supporting Information

Physical Characterization of Novel, Bioinspired Glycolipid Surfactants

Tyler J. Durkin[1]*, Kelsey R. Graves[1], Suchol Savagatrup[2], Raina M. Maier[1], David E. Hogan[1]

[1] Department of Environmental Science, University of Arizona,

1177 E 4th St, Tucson, AZ, 85719

[2] Department of Chemical and Environmental Engineering, the University of Arizona

1133 E. James E. Rogers Way, Tucson Arizona 85721

* Authors to whom correspondence should be addressed: tydurkin@arizona.edu

S.1 Critical Micelle Concentration Calculation of All Surfactants

Calculation of the critical micelle concentration using a surface tension vs. concentration plot is detailed fully in the manuscript using Rha-C10 as an example. Similar surface tension vs. concentration plots were prepared for each of the other head and tail group combinations (Rha-C12 through C18, Rha-C10-C10 through C14-C14, as well as Xyl- and Gal- C10 through C18) and are included below.

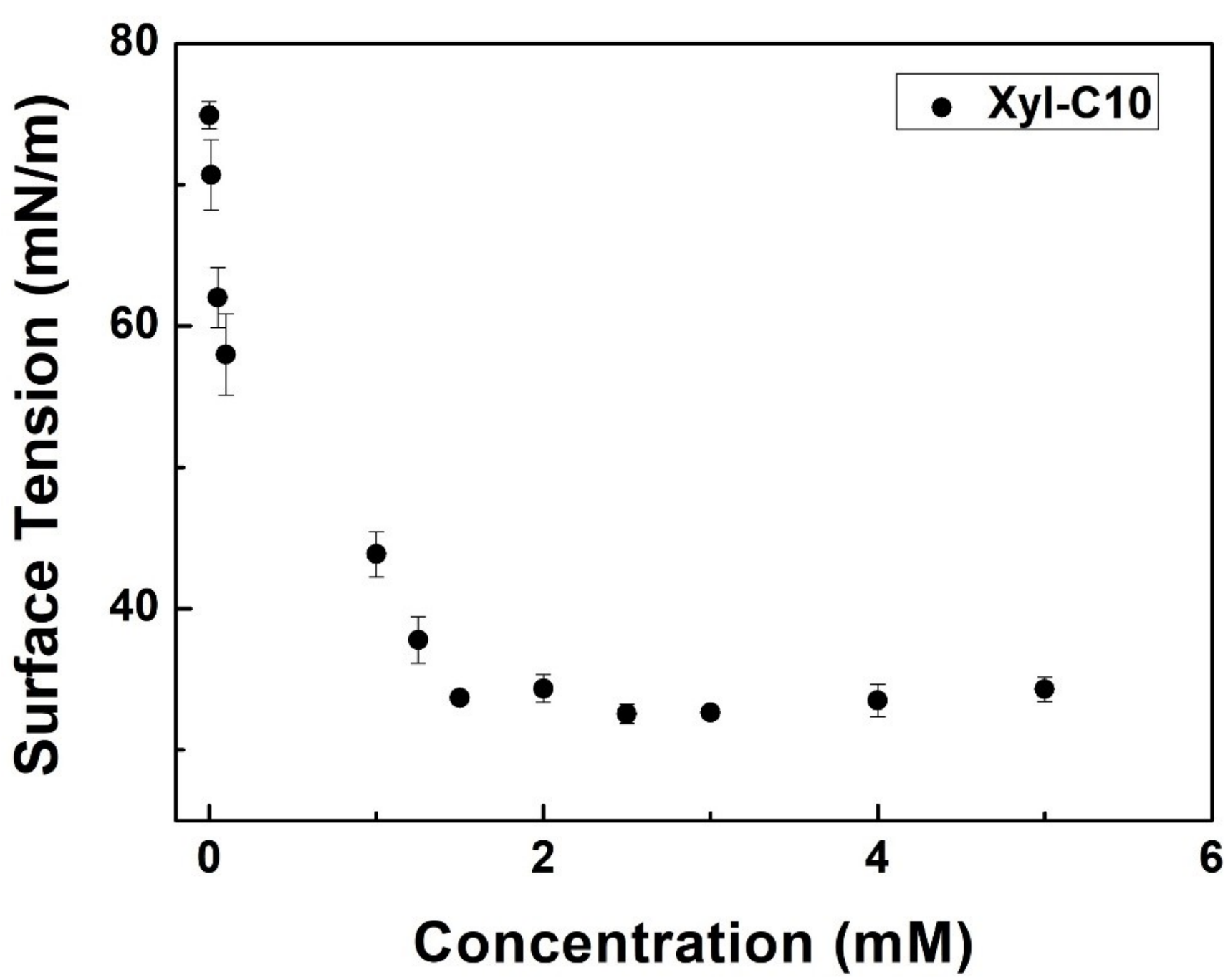

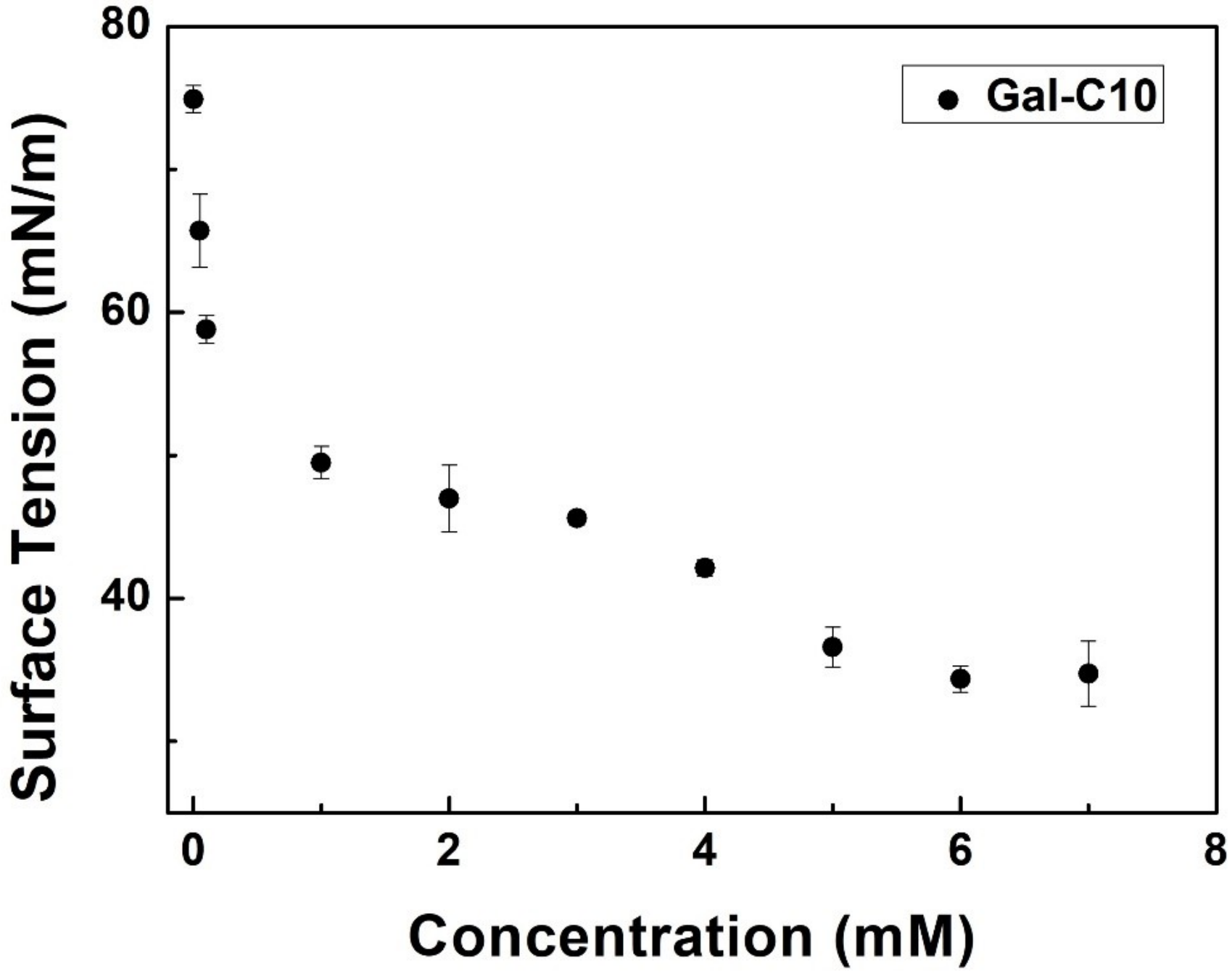

Gal-C10
Surface Tension (mN/m)
Concentration (mM)
80
60
40
0
2
4
6
8

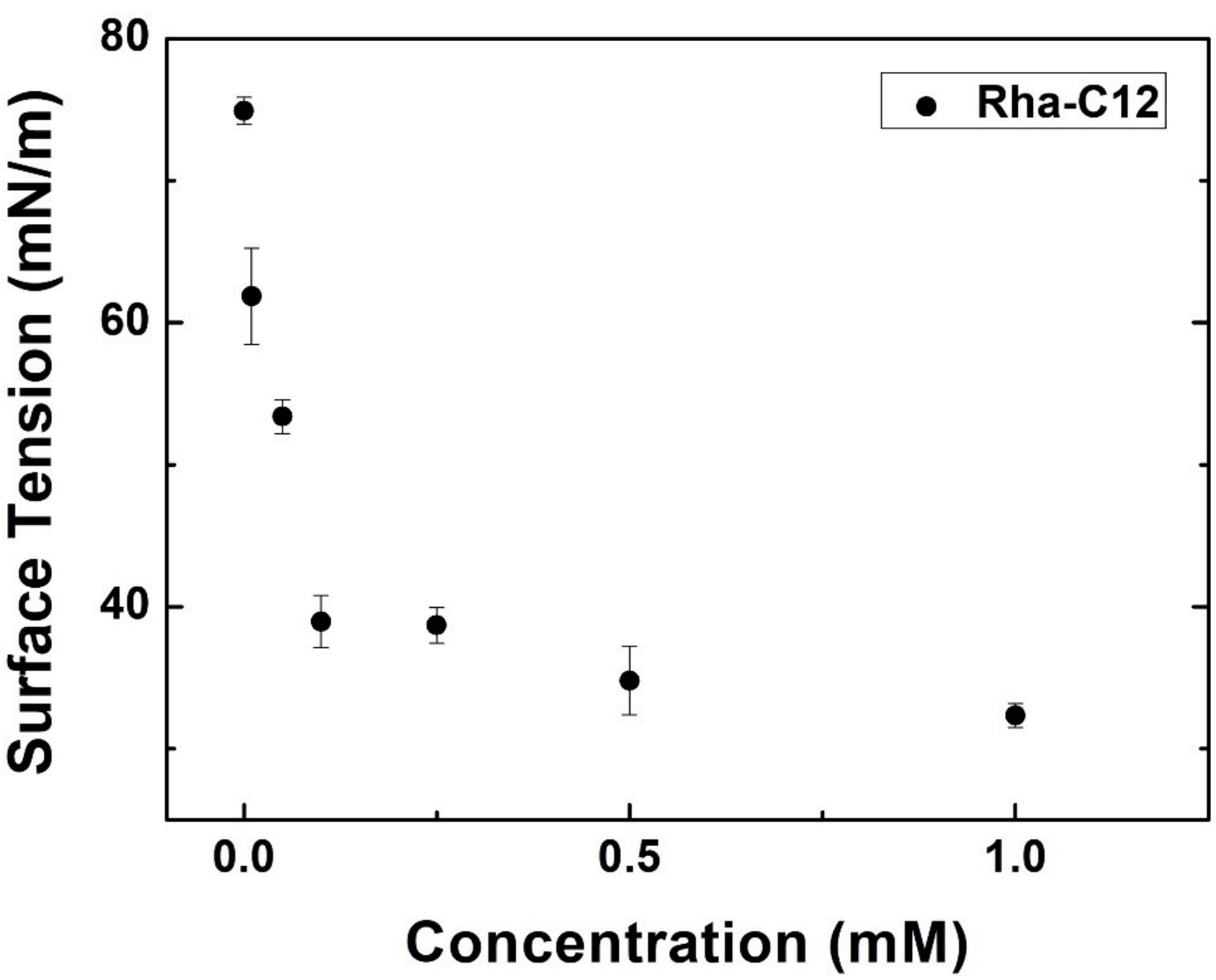

Rha-C12
Surface Tension (mN/m)
Concentration (mM)
80
60
40
0.0
0.5
1.0

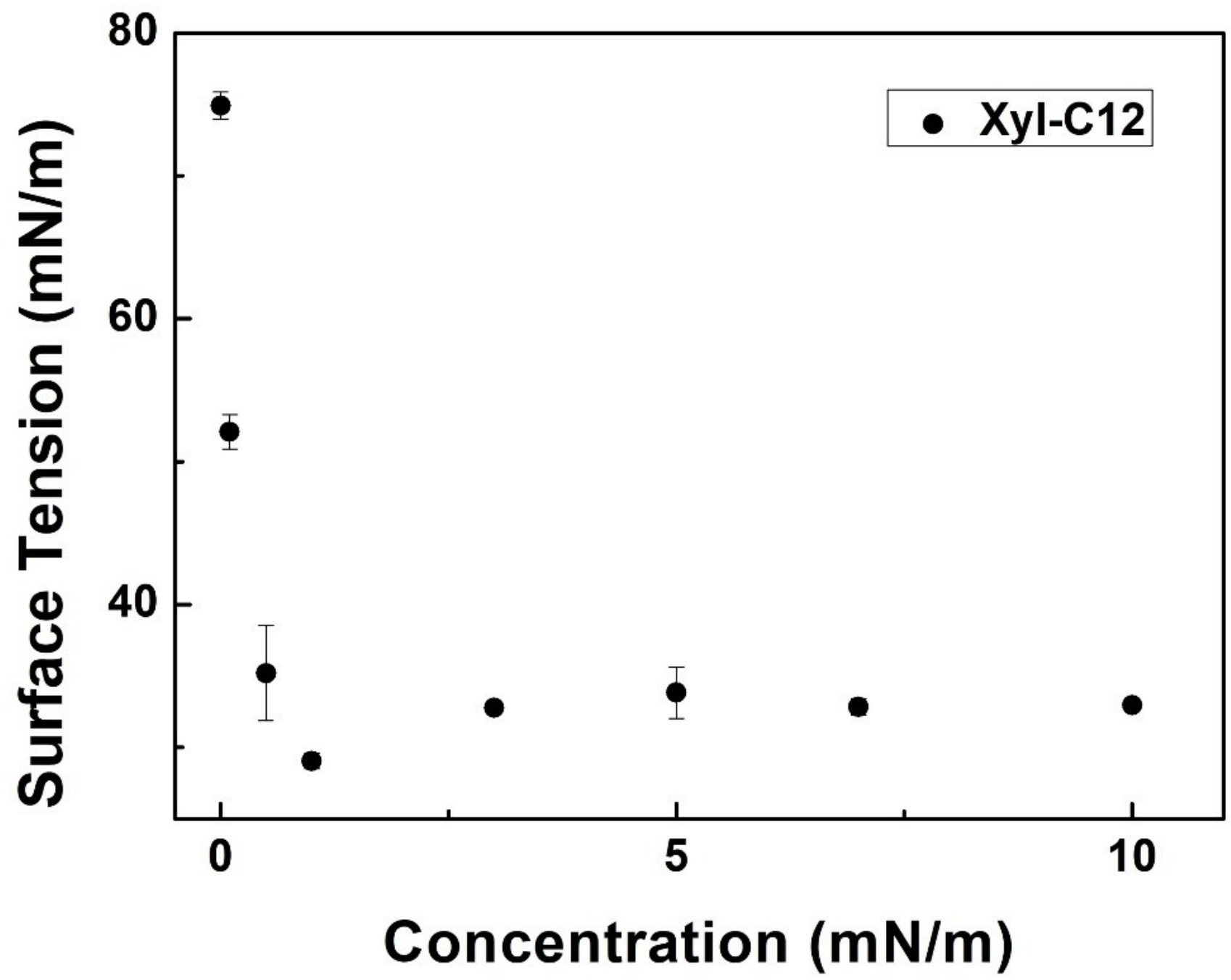

80
60
40
Surface Tension (mN/m)
Xyl-C12
0
5
10
Concentration (mN/m)

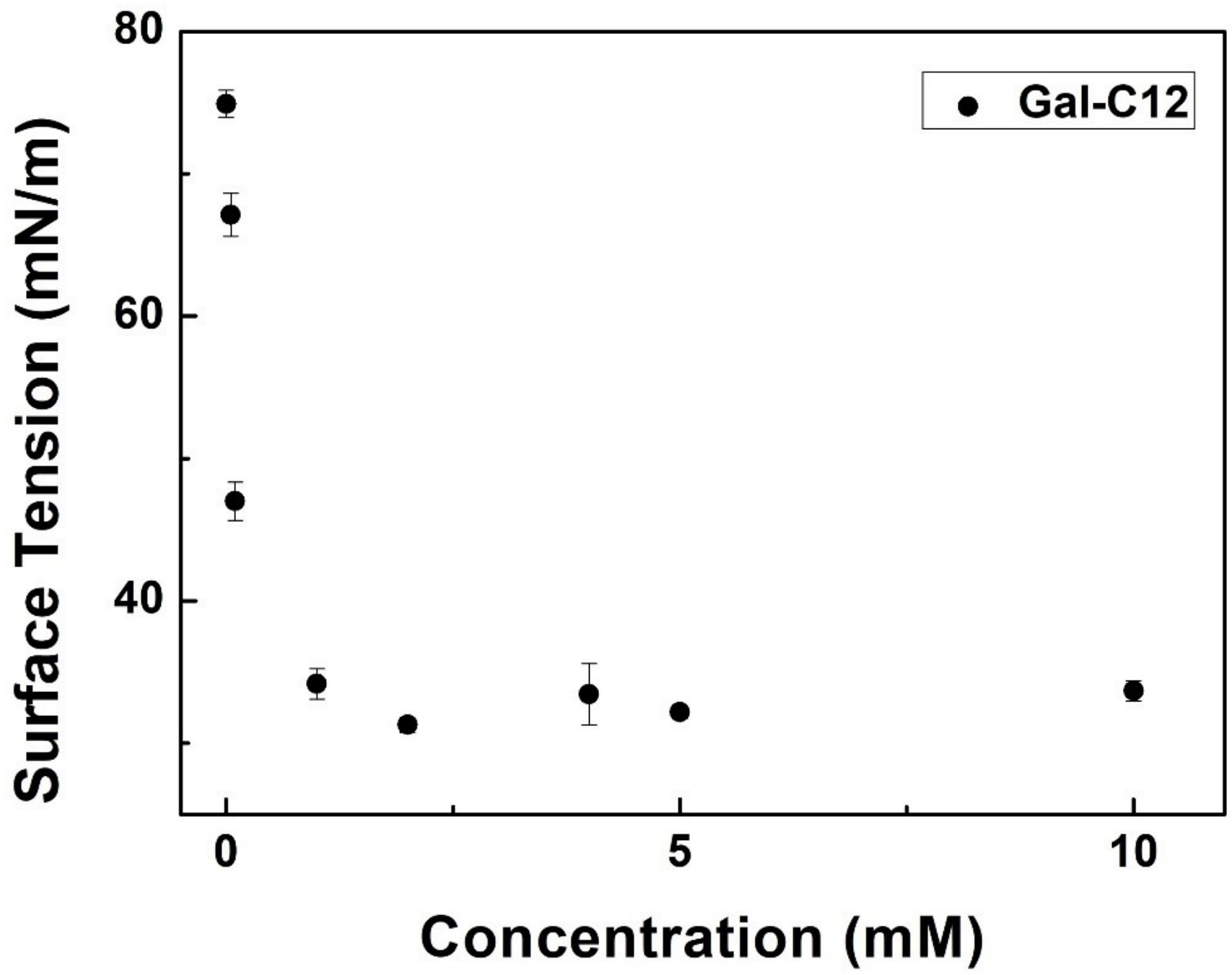

80
60
40
Surface Tension (mN/m)
Gal-C12
0
5
10
Concentration (mM)

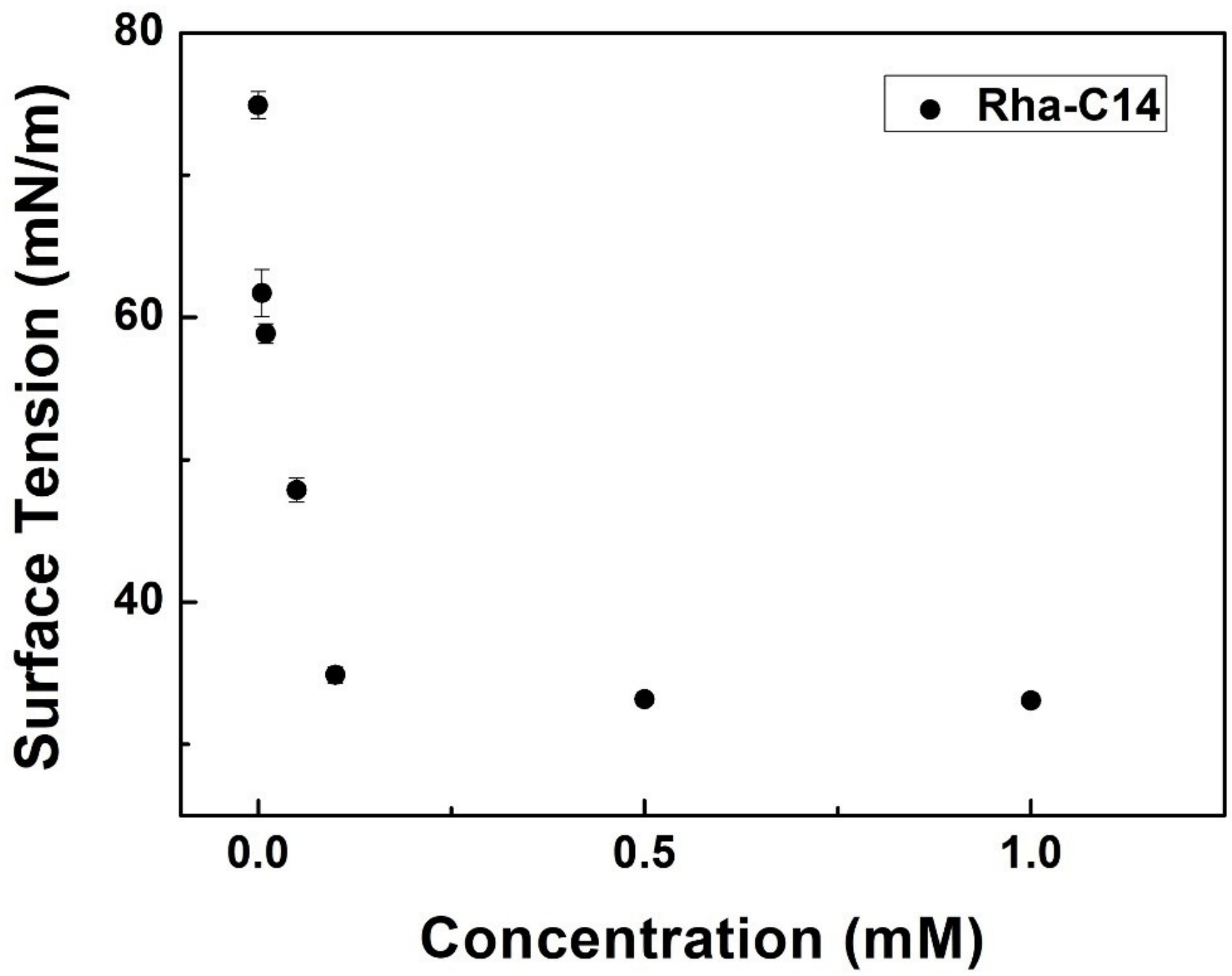

Surface Tension (mN/m)
80
60
40
Rha-C14
0.0
0.5
1.0
Concentration (mM)

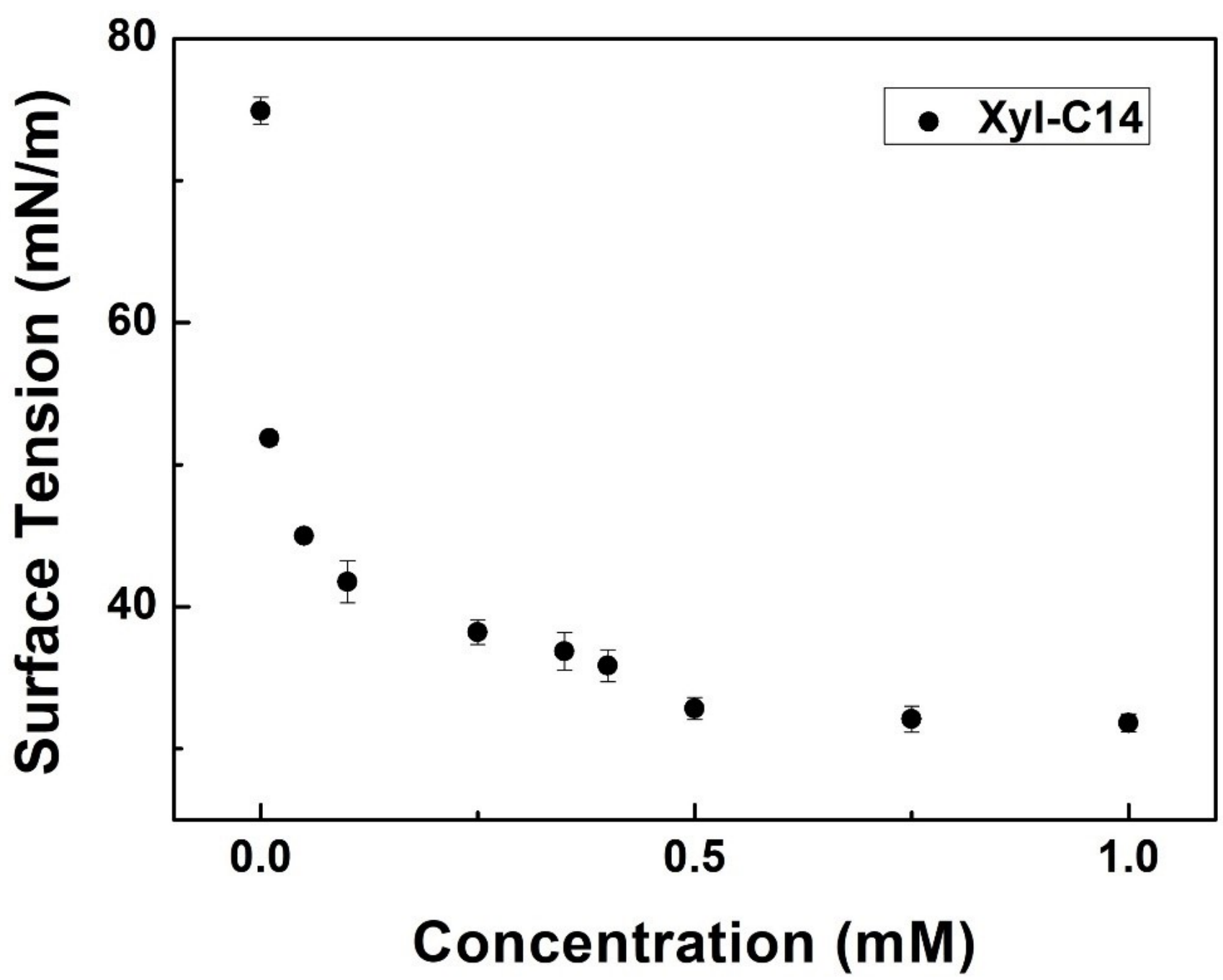

Surface Tension (mN/m)
80
60
40
Xyl-C14
0.0
0.5
1.0
Concentration (mM)

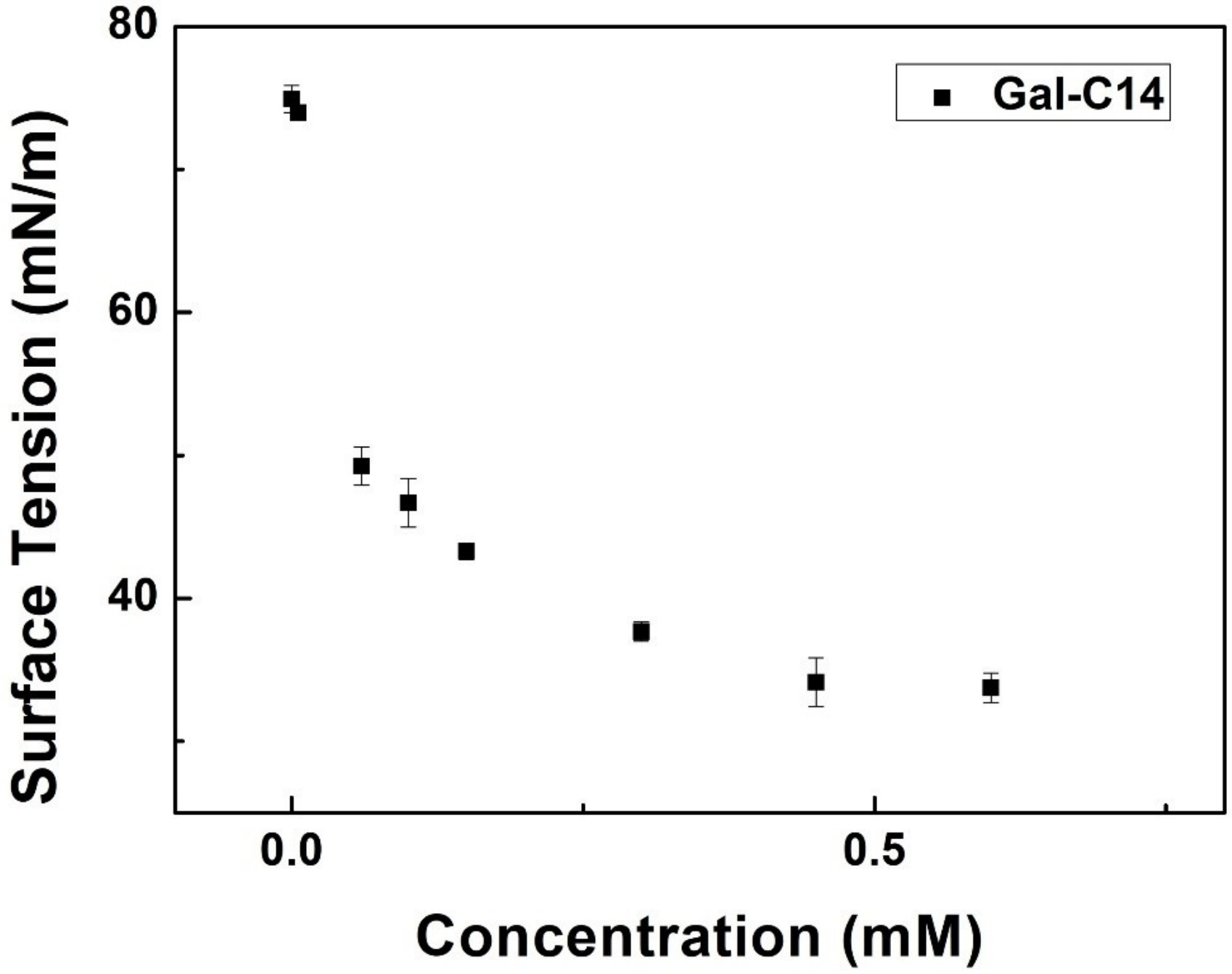
Gal-C14
Surface Tension (mN/m)
80
60
40
0.0
0.5
Concentration (mM)

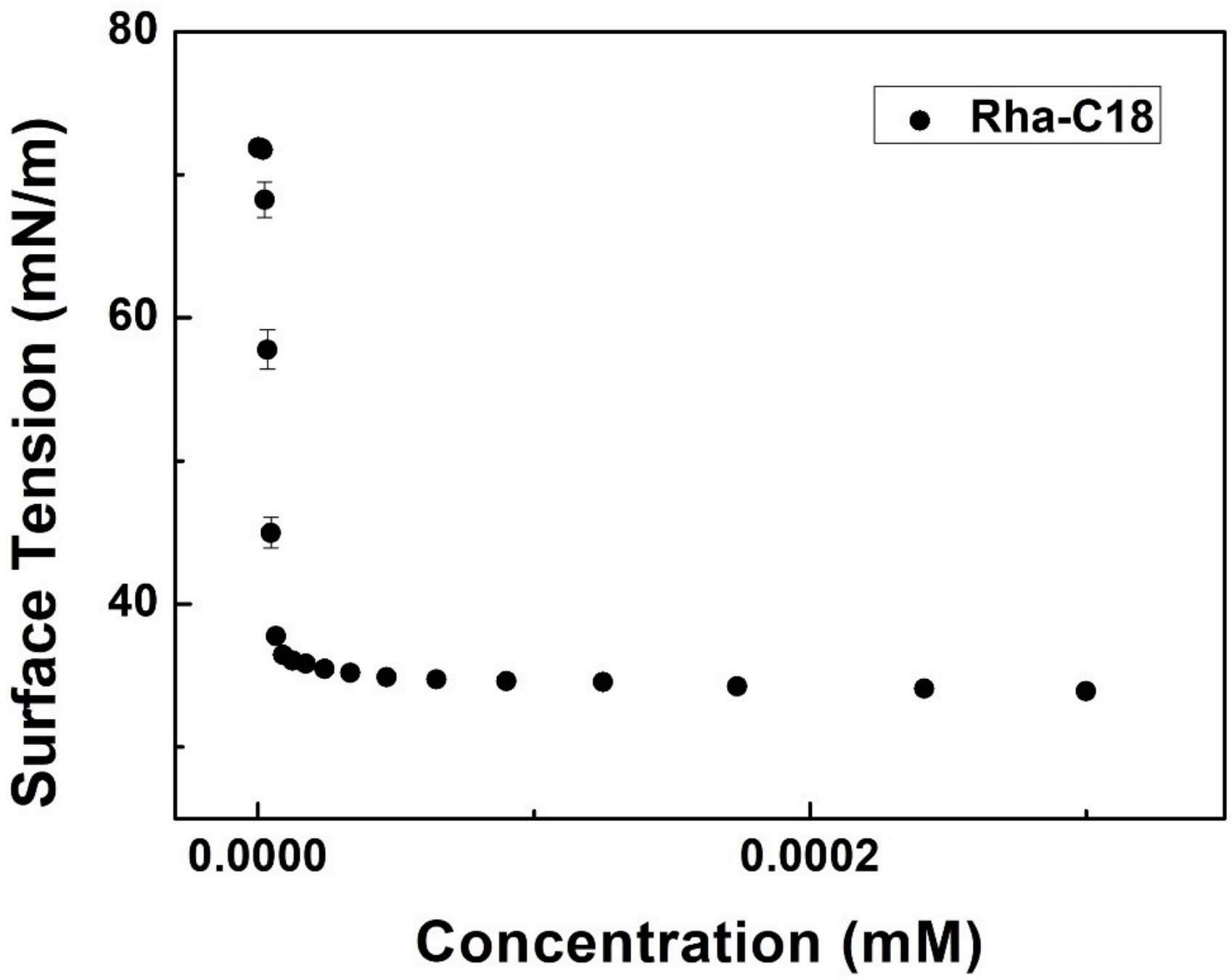
Rha-C18
Surface Tension (mN/m)
80
60
40
0.0000
0.0002
Concentration (mM)

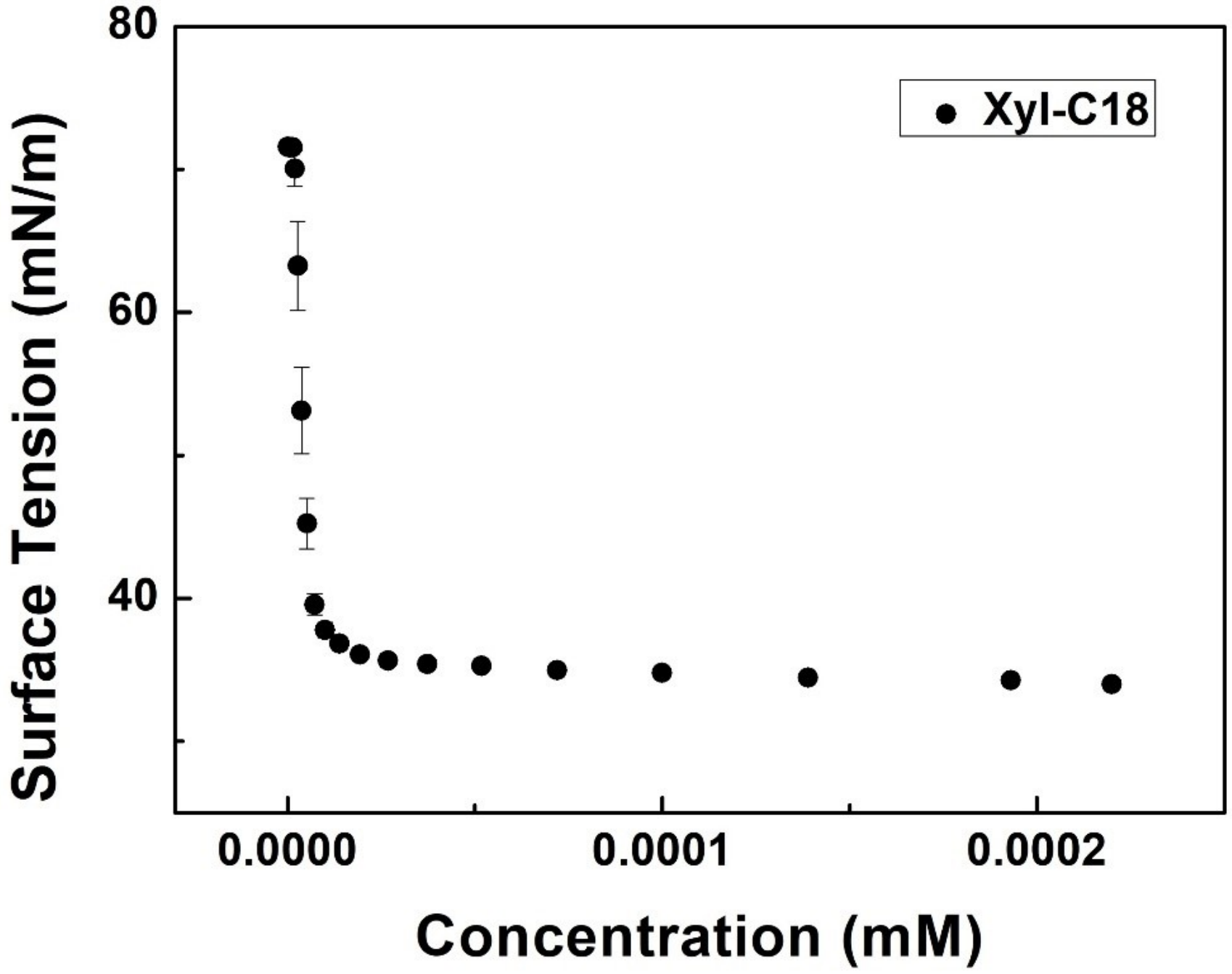
Xyl-C18
Surface Tension (mN/m)
Concentration (mM)
80
60
40
0.0000
0.0001
0.0002

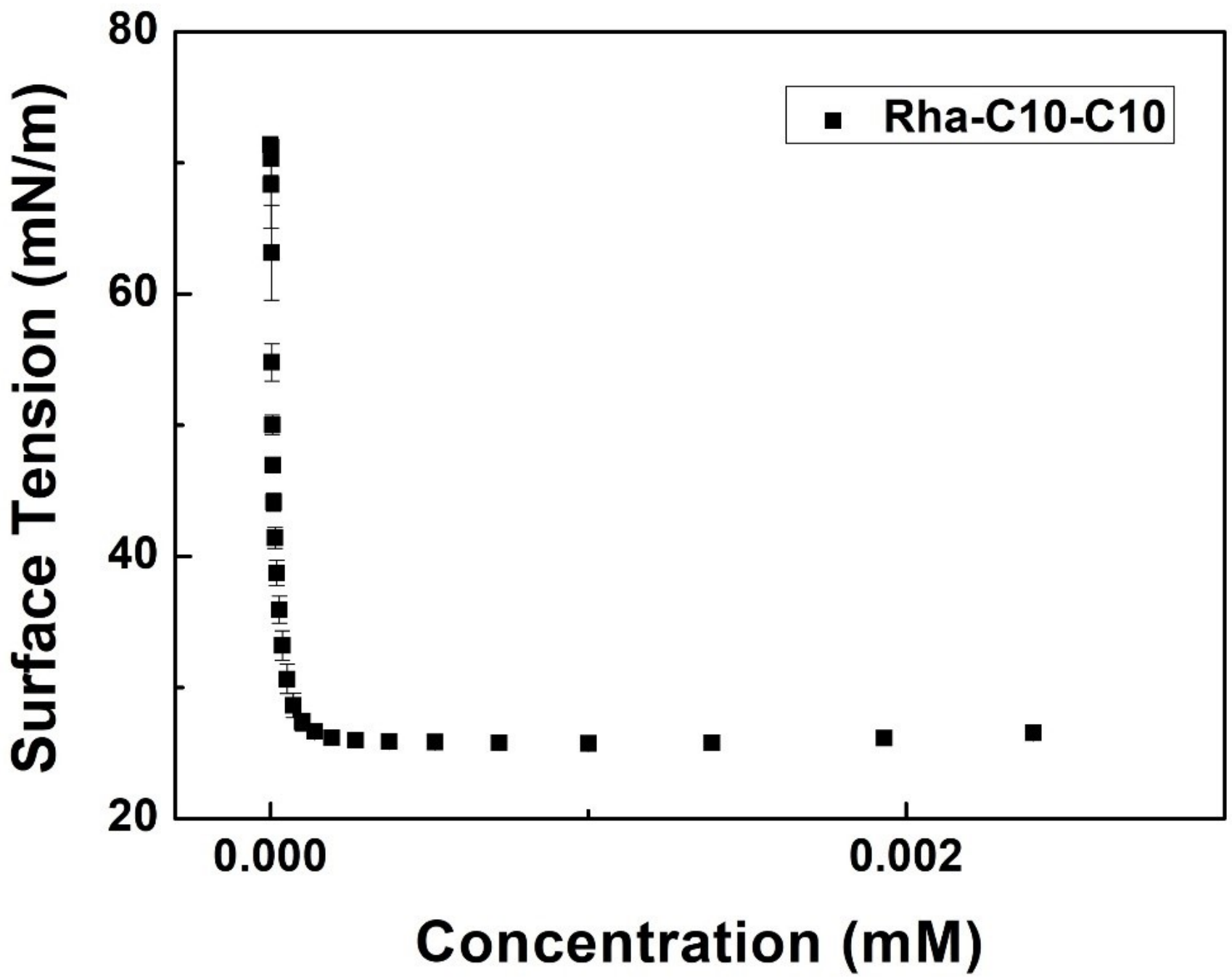
Rha-C10-C10
Surface Tension (mN/m)
Concentration (mM)
80
60
40
20
0.000
0.002

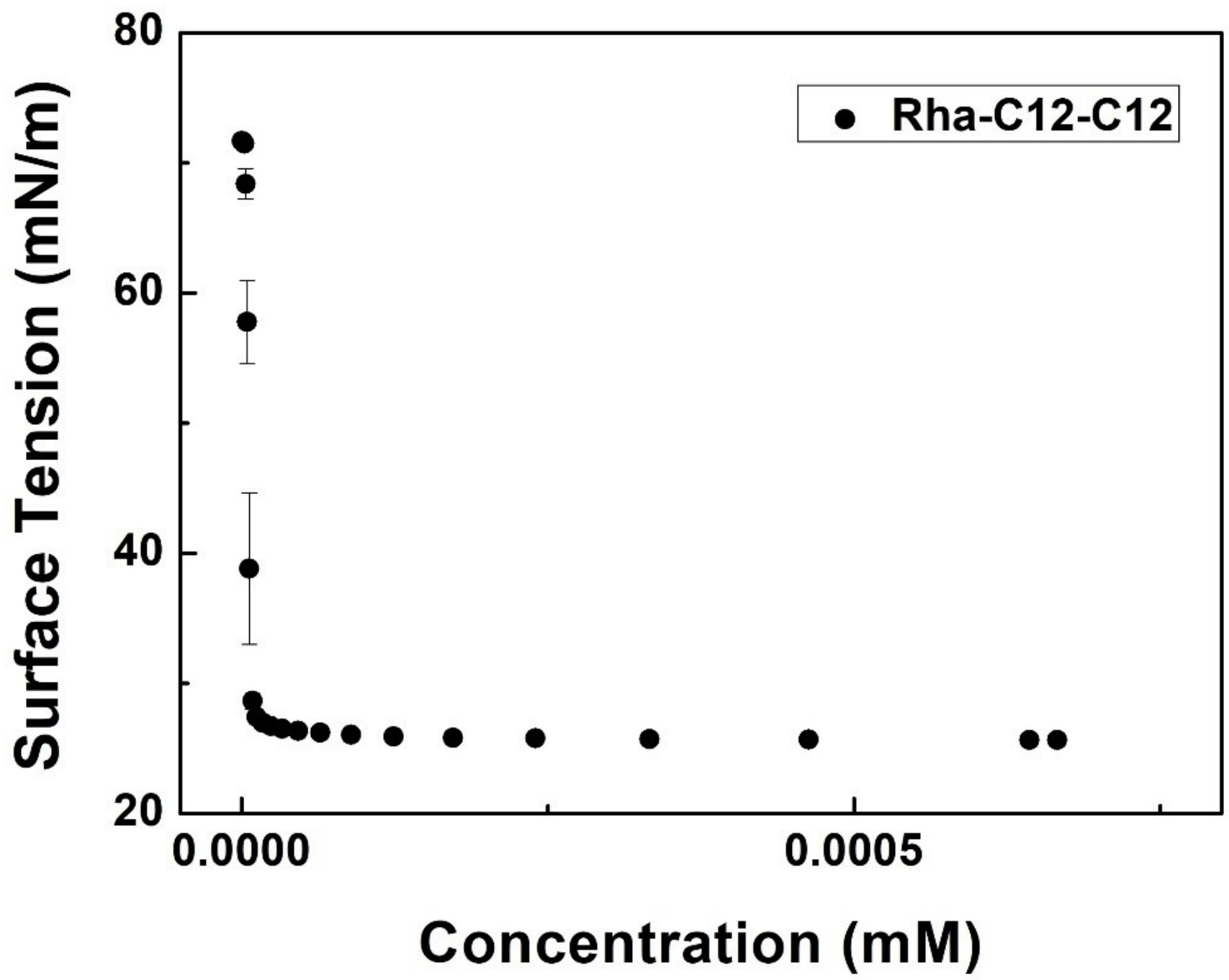
Rha-C12-C12
Surface Tension (mN/m)
80
60
40
20
0.0000
0.0005
Concentration (mM)

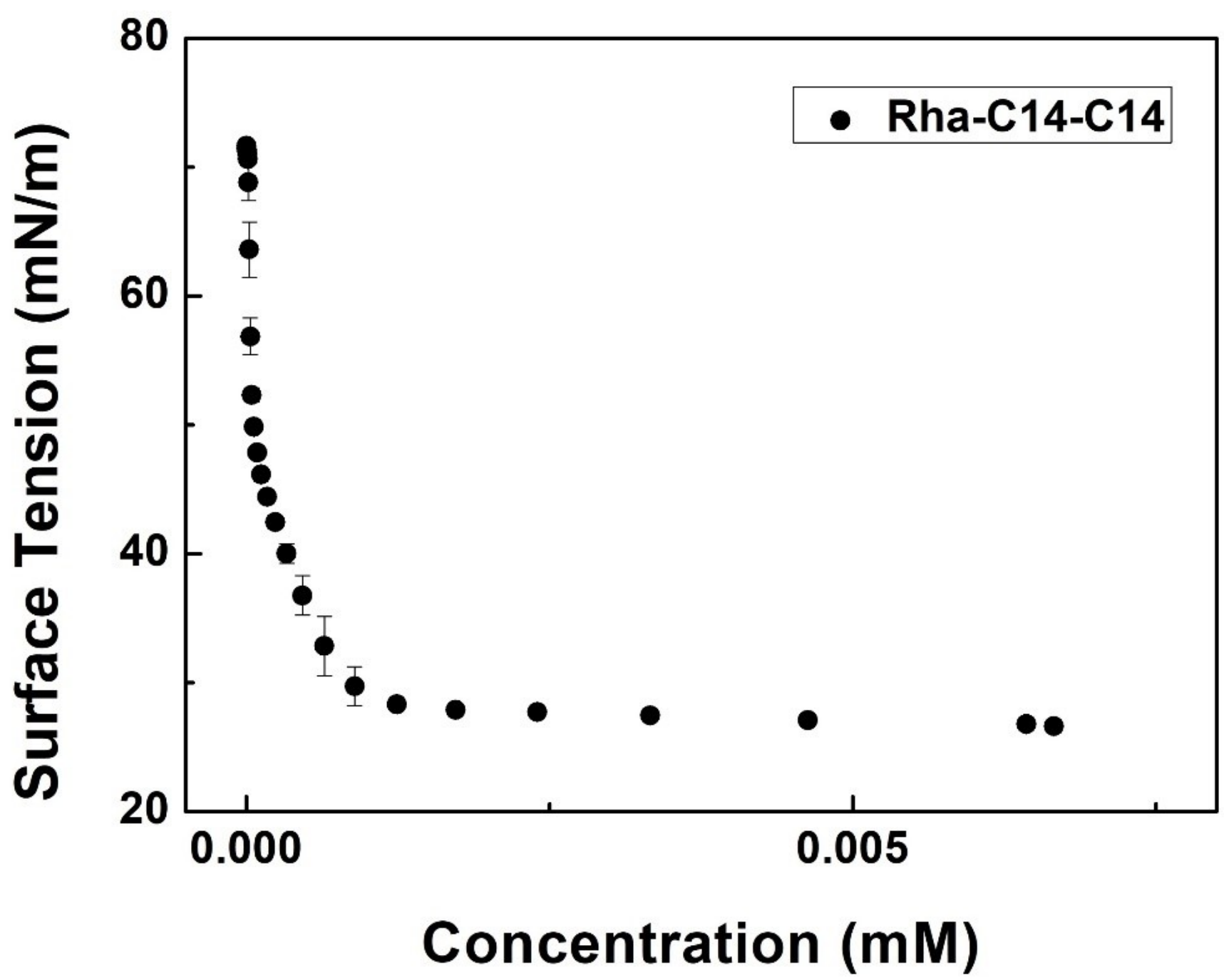
Rha-C14-C14
Surface Tension (mN/m)
80
60
40
20
0.000
0.005
Concentration (mM)

S.2
